# Development and Validation of an Artificial Intelligence Preoperative Planning System for Total Hip Arthroplasty

**DOI:** 10.3389/fmed.2022.841202

**Published:** 2022-03-22

**Authors:** Xi Chen, Xingyu Liu, Yiou Wang, Ruichen Ma, Shibai Zhu, Shanni Li, Songlin Li, Xiying Dong, Hairui Li, Guangzhi Wang, Yaojiong Wu, Yiling Zhang, Guixing Qiu, Wenwei Qian

**Affiliations:** ^1^Department of Orthopedic Surgery, Peking Union Medical College Hospital, Peking Union Medical College, Chinese Academy of Medical Sciences, Beijing, China; ^2^School of Life Sciences, Tsinghua University, Beijing, China; ^3^Institute of Biomedical and Health Engineering (iBHE), Tsinghua Shenzhen International Graduate School, Shenzhen, China; ^4^Department of Biomedical Engineering, School of Medicine, Tsinghua University, Beijing, China; ^5^Longwood Valley Medical Technology Co. Ltd., Beijing, China; ^6^School of Medicine, Tsinghua University, Beijing, China; ^7^Department of Orthopedics, Beijing Tongren Hospital, Capital Medical University, Beijing, China; ^8^Peking Union Medical College Hospital, Peking Union Medical College, Chinese Academy of Medical Sciences, Beijing, China; ^9^Department of Plastic Surgery, Sichuan University West China Hospital, Chengdu, China

**Keywords:** arthroplasty, artificial intelligence, hip, convolutional neural network, preoperative planning

## Abstract

**Background:**

Accurate preoperative planning is essential for successful total hip arthroplasty (THA). However, the requirements of time, manpower, and complex workflow for accurate planning have limited its application. This study aims to develop a comprehensive artificial intelligent preoperative planning system for THA (AIHIP) and validate its accuracy in clinical performance.

**Methods:**

Over 1.2 million CT images from 3,000 patients were included to develop an artificial intelligence preoperative planning system (AIHIP). Deep learning algorithms were developed to facilitate automatic image segmentation, image correction, recognition of preoperative deformities and postoperative simulations. A prospective study including 120 patients was conducted to validate the accuracy, clinical outcome and radiographic outcome.

**Results:**

The comprehensive workflow was integrated into the AIHIP software. Deep learning algorithms achieved an optimal Dice similarity coefficient (DSC) of 0.973 and loss of 0.012 at an average time of 1.86 ± 0.12 min for each case, compared with 185.40 ± 21.76 min for the manual workflow. In clinical validation, AIHIP was significantly more accurate than X-ray-based planning in predicting the component size with more high offset stems used.

**Conclusion:**

The use of AIHIP significantly reduced the time and manpower required to conduct detailed preoperative plans while being more accurate than traditional planning method. It has potential in assisting surgeons, especially beginners facing the fast-growing need for total hip arthroplasty with easy accessibility.

## Highlights

### Article Focus

– Develop an artificial intelligent preoperative planning system for THA (AIHIP) with increased inefficiency.
– Conduct a prospective clinical study to validated the efficacy of AIHIP.

### Key Messages

– Convolutional neural networks automated the processing of CT images and achieved satisfactory accuracy.
– AIHIP significantly reduced the time and manpower required to conduct detailed preoperative planning.

## Background

Total hip arthroplasty (THA) is the primary surgical procedure performed for the treatment of pain and impaired function associated with osteoarthritis, osteonecrosis, fracture and other diseases. It is among the top 5 most commonly performed procedures and the top 5 fastest growing procedures (1). By 2030, the annual counts of THA are estimated to be 572–1,385 thousand (2).

The goals of THA are to minimize discomfort, improve hip function and prolong implant survival. However, leg length discrepancy (LLD), dislocation and implant failure remain primary challenges to success. Accurate preoperative planning may help surgeons achieve successful THA because it provides a detailed assessment of preoperative deformities, predicts implant sizes, provides intraoperative references and simulates postoperative outcomes such as leg length (3). Preoperative planning based on X-ray remains one of the most common methods. However, the accuracy of X-ray-based planning is controversial, ranging from 40.7 to 99.2% (3–7). Many factors may limit the accuracy of X-ray-based planning: surgeon experience, magnification error, patient position and the nature that X-ray images can only provide 2-dimensional information (8).

CT-based preoperative planning offers more detailed information on a three-dimensional scale (9). However, CT-based planning requires a complex workflow that includes image segmentation, pelvis correction, deformity recognition and postoperative simulation. Therefore, the application of CT-based planning systems is limited in that they are especially time-consuming for each case and require a group of experienced engineers, programmers and doctors to work closely together (10).

Artificial intelligence (AI) techniques, including convolutional neural networks (CNNs), have shown promising results in processing medical images with high accuracy and significantly reduced time requirements (11, 12). However, the clinical application of artificial intelligence has mainly focused on diagnosing diseases (13–15). Research to date has not yet validated the use of AI in preoperative planning systems for THA.

With the help of artificial intelligence, it is possible to develop efficient and accurate preoperative THA planning system. The purpose of this study is described as follows. 1. A comprehensive artificial intelligent preoperative planning system for THA (AIHIP) was developed, which included automatic image segmentation, preoperative deformity recognition and real-time postoperative outcome simulation. 2. A prospective clinical study was conducted to compare the efficacy between AIHIP planning and X-ray-based planning.

## Methods

### The Primary Development Goals for Artificial Intelligent Preoperative Planning System

**FIGURE 1:**
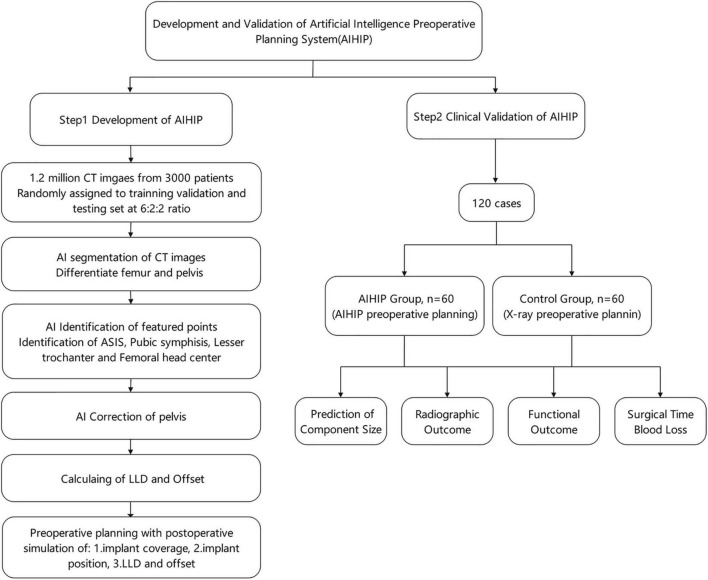
Flow chart of the development and clinical validation of artificial intelligence preoperative planning system for THA (AIHIP).

The AIHIP planning system included preoperative assessment and postoperative outcome simulations. Image segmentation was required to differentiate the femur from the pelvis. Featured anatomic landmarks were identified to serve as references. Then, the pelvis was corrected to a neutral position in the sagittal and coronal planes according to the identified landmarks. Assessment of preoperative deformity was also conducted referring to the identified landmarks. Postoperative outcome simulations showed the planned implant size, implant coverage and to what degree the preoperative deformity could be corrected. The comprehensive workflow was integrated into the AIHIP software. The accuracy of the AIHIP was validated through a clinical study. The research workflow is shown in Figure 1.

### Data Acquisition

Over 1.2 million qualified CT images from 3,000 anonymized patients were included in this study. All patients were scheduled to receive total hip arthroplasty. The primary diagnosis included osteonecrosis, osteoarthritis, rheumatoid arthritis and developmental dysplasia of the hip. Standardized pelvic CTs were conducted prior to the operation. The range of each CT scan began from the highest point of the pelvis to 15 cm below the lesser trochanter at 1 mm intervals. All CTs were stored according to the DICOM protocol.

### Ground Truth Definition

CT images were manually segmented by a group of engineers and three orthopedic surgeons using Mimics Software (Materialise NV, Leuven, Belgium). All engineers and surgeons had performed manual segmentation for at least 50 cases prior to this study. The contours of the femur and pelvis were manually annotated. Featured anatomic landmarks were manually marked, which included the anterior superior iliac spine (ASIS), pubic symphysis, center of the femoral head, medial edge of the lesser trochanter, and anatomic axis of the femur.

### Image Segmentation Module

**FIGURE 2:**
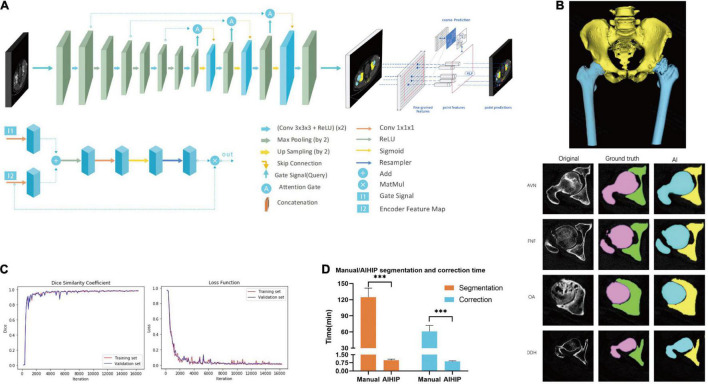
Development of artificial intelligence preoperative planning system for THA (AIHIP): image segmentation. **(A)** Net-work structure; **(B)** segmentation of pelvis and femur. Images of original CT, manual segmentation, and automatic segmentation with AIHIP in four primary diseases: avascular necrosis (AVN), femoral neck fracture (FNF), osteoarthritis (OA), and developmental dysplasia of hip (DDH). 3D reconstruction of the CT was completed after segmentation; **(C)** performance of AIHIP in automatic segmentation. Dice similarity coefficient (DSC) of training set and validation set. Loss of training set and validation set; **(D)** time comparison between manual segmentation and artificial intelligence (AI) segmentation. Time comparison between manual correction and AI correction. ***p < 0.001.

The complete dataset was randomly assigned to a training set, validation set and testing set at a ratio of 6:2:2. All images were resized to 512 × 512 pixels. The neural network structure was developed based on the attention U-Net with a point rend module. The U-Net convolutional neural network can automatically segment CT images and has achieved high accuracy in recognizing abdominal organs and tissues (16). The point rend module was used to provide point-based predictions to further enhance segmentation performance (17). An attention U-Net was developed based on a U-Net with added skip connections and attention gates (18). The use of an attention gate enables the network to automatically focus on target structures without requiring large computational power and model parameters. A skip connection was conducted between the corresponding encoder and decoder layers. Implementing skip connections provided segmentation results with a higher level of accuracy because more graphic features from the basic level were preserved and integrated into the output feature. The increased number of decoders also provides a more detailed segmentation (Figure 2A). The Dice similarity coefficient (DSC) and loss was used to assess the model performance of the AIHIP in the segmentation of CT images. DSC and loss were calculated for every 100 iterations.

### Identification of Featured Anatomic Landmarks

**FIGURE 3:**
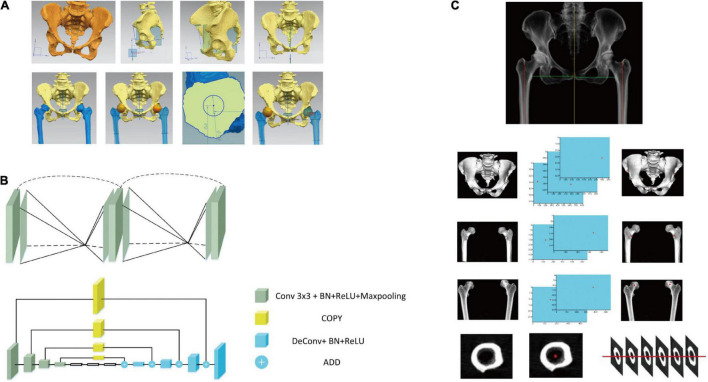
Development of artificial intelligence preoperative planning system for THA (AIHIP): correction of pelvis, identification of anatomical landmarks and recognition of preoperative deformities. **(A)** Manual correction and measurement of pelvis and femur; **(B)** network structure used to identify featured anatomic landmarks; **(C)** examples of automatic identification of anterior superior iliac spine (ASIS), medial point of lesser trochanter and center of femoral head. The anatomic axis of femur was identified using least square method.

An example of manual identification of feature anatomic landmarks and correction of pelvis is shown in Figure 3A. Based on the segmented pelvis, the automatic recognition of featured anatomic landmarks was conducted with a stacked hourglass network, which has been used in human pose estimation (19). Repeated bottom-up and top-down inference is beneficial in predicting the coordinates of the featured anatomic structure. The stacked hourglass network automatically recognized featured anatomic structures, including the bilateral anterior superior iliac spine (ASIS), the pubic symphysis and the center of the femoral head (Figure 3B). The least square method was used to determine the anatomic axis of the femur. The pelvis could be adjusted, and the preoperative deformity could be measured based on these identified points (Figure 3C).

### Preoperative Planning Module

**FIGURE 4:**
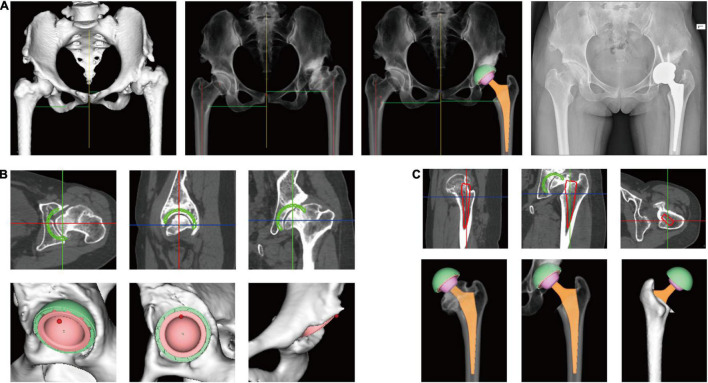
Preoperative planning using artificial intelligent preoperative planning system for THA (AIHIP). **(A)** From left to right: 3D reconstructed pelvis and femur; simulated hip X-ray; simulated postoperative outcome; postoperative X-ray; **(B)** preoperative planning of acetabular component. The green circle shows the planned component position in real-time. Bone coverage was calculated once the size, position, inclination, and anteversion of acetabular component is determined; **(C)** preoperative planning of femoral component. The red circle shows the planned position of femoral component in real-time.

Preoperative planning was conducted by two orthopedic surgeons using AIHIP software (Version 3.0, Longwood Valley Technology, China). Planning was carried out by first determining the position and size of the acetabular component. Inclination, anteversion, and coverage of the acetabular component were planned as well. Then, the position and size of the femoral component were determined, and the level of femoral resection was determined. Different types of acetabular components, femoral components, and femoral heads could be chosen. A simulation of the postoperative effect was generated, which showed the postoperative leg length, offset and coverage of the acetabular component. Leg length and offset of the contralateral side were also shown so that changes in the surgical plan could be made accordingly. The distance between the tip of the lesser trochanter and the tip of the femoral stem (neck length) and the distance between the tip of the lesser trochanter and the resection line of the femoral neck (calcar length) were measured in preoperative planning as references. Then, the operating surgeon measured the neck length and calcar length intraoperatively for verification (Figures 4A–C).

### Clinical Validation of Artificial Intelligent Preoperative Planning System for THA

Approval from the Institutional Review Board and written informed consent was acquired to conduct a prospective clinical study from October 2019 to February 2021. Patients were included if they 1. were diagnosed with osteonecrosis, osteoarthritis and developmental dysplasia of the hip (Crowe I) and received THA; 2. provided written informed consent to participate in the study. Patients were excluded if 1. the preoperative or postoperative radiographs were not standardized or 2. different types of prostheses were used during surgery. A total of 120 cases were included. X-ray-based planning was conducted in 60 cases (control group) according to the method described by Della Valle et al. (3), where planning was completed directly over the printed X-ray using templates. AIHIP planning was conducted in 60 cases (AIHIP group).

Leg length discrepancy, offset, neck length, and calcar length were measured on postoperative radiographs with the patients’ names concealed. Each measurement was made and recorded by the two observers at least 4 weeks after the operation to avoid any recollection bias. The mean value of the two measurements was used for statistical analysis. Inter-observer reliability of radiographic measurement was assessed with intraclass correlation coefficient (ICC).

Functional outcome was assessed by Hip Disability and Osteoarthritis Score Joint Replacement (HOOS JR) (20) and EuroQol 5 Dimensions Questionnaire (EQ-5D) (21, 22). Patients were followed up until 12 weeks postoperatively. Surgical time and blood loss were also recorded.

### Surgical Technique and Peri-Operative Management

All patients underwent total hip arthroplasty through a posterior approach by one experienced orthopedic surgeon in one facility. The prostheses used were Pinnacle Cup (DePuyOrthopaedics, Warsaw, IN, United States), Corail Stem (DePuyOrthopaedics, Warsaw, IN, United States) and Trilock stem (DePuyOrthopaedics, Warsaw, IN, United States). Standard perioperative care and patient education were administered to all patients.

### Statistical Analysis

Statistical analysis was performed with SPSS version 25 (IBM, New York, NY, United States) and GraphPad Prism version 8 (GraphPad Software, San Diego, CA, United States). According to previous literatures (23–25), accurate prediction was defined as the predicted size to be within ± 1 size from the implanted size. Absolute error was defined as the absolute difference between planned and implanted component size and the difference between plan and postoperative radiographic measurement. Mean error was defined as the average value of the planned component size minus the implanted component size. A *p*-value less than 0.05 was considered statistically significant. Discontinuous variables were recorded as incidence and rate. The chi-square test was used to compare the discontinuous variables between groups. Continuous variables were recorded as the means and standard deviation. A general linear model was used to test whether there was a statistically significant difference between the two groups considering confounding factors, including age, sex, BMI, and primary diagnosis.

## Results

### Validation of Artificial Intelligent Algorithms

The effect of manual segmentation and AI segmentation from four common primary diagnoses are shown in Figure 2B. The DSC curves and loss from the training set and validation set are shown in Figure 2C. Both curves reached convergence by 15,800 iterations, which indicated optimal DSC and loss. At 15,800 iterations, the DSC of the training set was 0.983, and the DSC of the validation set was 0.987. The losses were 0.008 and 0.013 for the training set and validation set, respectively (Figure 2C). The testing set was used to validate algorithm performance. The testing set achieved a DSC of 0.973 and loss of 0.012, which was comparable to that of the training set and validation set.

The average time consumption was 0.99 ± 0.94 min for AIHIP segmentation and 0.87 ± 0.07 min for AIHIP correction and deformity assessment. The average time was 124.55 ± 16.87 min for manual segmentation and 60.85 ± 11.11 min for manual correction and deformity assessment (Figure 2D). The total time required for the AIHIP algorithm to process the CT for one case was 1.86 ± 0.12 min on average, compared with 185.40 ± 21.76 min for the manual workflow (*P* < 0.001).

### Clinical Validation

A total of 120 cases were included in the study. The demographic characteristics are listed in Table 1, which include age, sex, height, weight, BMI, and primary diagnosis. The difference in age between the control group (mean: 53.75, range: 24–78) and the AIHIP group (mean: 47.62, range: 23–78) was statistically significant (*P* = 0.033). There were no statistically significant differences in terms of sex, weight, height, or BMI. Demographic characteristics were considered as confounding variables. Their influences on the prediction accuracy were assessed and adjusted with generalized linear models.

**TABLE 1:** Demographic characteristics.

	AIHIP (*n* = 60)		Control (*n* = 60)		
		
	Mean	Std	Mean	Std	*P*-value
Height (cm)	165.15	8.04	165.98	8.03	0.571
Age (years)	47.62 (range: 23–78)	15.30	53.75 (range: 24–78)	16.10	0.033
Weight (kg)	66.12	10.58	69.29	11.77	0.123
BMI (kg/m^2^)	24.19	3.08	25.14	3.78	0.134
Gender	Male = 29, Female = 31		Male = 32, Female = 28		0.584
**Primary disease**					
Osteonecrosis	*n* = 41		*n* = 33		0.467
DDH(Crowe I)	*n* = 10		*n* = 11		
Osteoarthritis	*n* = 3		*n* = 7		
Old Fracture	*n* = 2		*n* = 1		
Ankylosing spondylitis	*n* = 4		*n* = 7		
Rheumatoid arthritis	*n* = 0		*n* = 1		

**FIGURE 5:**
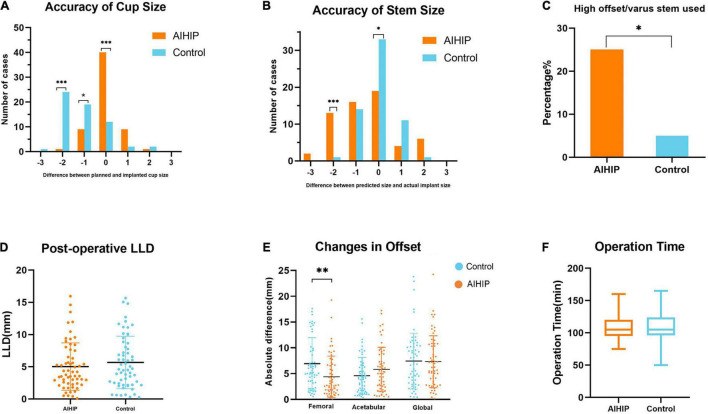
Clinical validation of artificial intelligent preoperative planning system for THA (AIHIP). **(A)** Plan accuracy of cup size; **(B)** plan accuracy of stem size; **(C)** proportion of high offset/varus stem used; **(D)** postoperative leg length discrepancy (LLD); **(E)** difference between preoperative and postoperative offset; **(F)** operation time. *, **, *** *P* < 0.05, 0.01, 0.001.

The predicted cup size and implanted cup size were exactly the same in 66.67% of the AIHIP cases and 20% of the control cases (*P* < 0.001). For femoral stem, the exact same size was achieved in 55% of the AIHIP cases and 31.67% of the control cases. The cup size was accurately predicted to within ± 1 size in 55.00 and 96.67% of patients in the control group and AIHIP group, respectively (*P* < 0.001). Stem size was accurately predicted to within ± 1 size in 65.00 and 96.67% of the control group and AIHIP group, respectively (*P* < 0.001). Further analysis showed more detailed comparison in Figures 5A, B. The tendency toward overestimation or underestimation of component size was assessed by mean error. Comparing with AIHIP planning, acetate templating tended to underestimate cup size by 2.13 ± 2.11 (*P* < 0.001) and underestimate the stem size by 0.53 ± 1.28 (*P* = 0.013). The comparison of the mean absolute error between the two groups is shown in Table 2. Compared with the control group, high offset/varus stems were more commonly used in the AIHIP group (*P* = 0.004) (Figure 5C). The average time it took to conduct X-ray planning was 7.37 ± 1.32 min and the average time it took to conduct AIHIP planning was 8.11 ± 0.98 min (*P* = 0.001).

**TABLE 2:** Accuracy of the surgical plan and radiographic outcome.

	AIHIP (*n* = 60)		Control (*n* = 60)		*P*-value
	
	Mean	Std	Mean	Std	
**Mean absolute error between preoperative planning and postoperative results**					
Cup size	0.73	1.10	2.53	1.6	<0.001
Stem size	0.48	0.57	1.07	0.88	<0.001
Neck length (mm)	5.49	4.40	6.13	3.16	0.813
Calcar length (mm)	3.92	2.79	4.51	2.96	0.249
**Changes between preoperative and postoperative offset**					
Femoral offset (mm)	4.41	3.99	6.91	5.08	0.001
Acetabular offset (mm)	5.83	4.29	4.59	3.55	0.163
Global offset (mm)	7.33	5.04	7.44	5.40	0.919
**Postoperative leg length discrepancy**					
LLD (mm)	5.03	3.67	5.68	4.06	0.360

*Confounding variables including age, gender, BMI and primary diagnosis were considered and none of them are factors with significant influence on the results.*

Neck length, calcar length, LLD, and offset were measured on radiographs. The difference between preoperative planning and postoperative radiographic measurements was recorded as the mean absolute error and is shown in Table 2. The ICCs for all radiographic measurement were above 0.9, which indicated substantial inter-observer agreement (Table 3). The mean absolute error for neck length was 6.13 ± 3.16 mm in the control group and 5.49 ± 4.40 mm in the AIHIP group (*P* = 0.813). The mean absolute error of calcar length was 4.51 ± 2.96 mm in the control group and 3.92 ± 2.79 mm in the AIHIP group (*P* = 0.249). The average postoperative LLD for the control group and AIHIP group was 5.68 ± 4.06 mm and 5.03 ± 3.67 mm, respectively (*P* = 0.360) (Figure 5D). Although a trend was observed in the above radiographic outcomes, the differences were not statistically significant. Changes in acetabular offset and global offset were not significantly different between the two groups. Femoral offset was more accurately restored in the AIHIP group than in the control group (*P* = 0.001) (Figure 5E).

**TABLE 3:** Inter-observer agreement of radiographic measurement.

	Calcar length	Neck length	LLD	Femoral offset		Acetabular offset	
					
				Preoperative	Postoperative	Preoperative	Postoperative
ICC	0.950	0.968	0.974	0.970	0.937	0.963	0.929

The average operation time was 106.83 ± 18.20 min in the AIHIP group and 109.58 ± 21.98 min in the control group (*P* = 0.457) (Figure 5F). The average blood loos was 285.00 ± 127.33 ml in the AIHIP group and 315.67 ± 164.68 ml in the control group (*P* = 0.256). There were no statistically significant differences in HOOS score preoperatively (*P* = 0.605) and 12 weeks postoperatively (*P* = 0.22) between the two groups. There were no statistically significant differences in EQ5D index preoperatively (*P* = 0.846) and 12 weeks postoperatively (*P* = 0.203) between the two groups.

## Discussion

We developed an artificial intelligence-based system (AIHIP) to enhance the efficiency and accuracy of preoperative planning for THA. Deep learning algorithms were used for the comprehensive workflow. In the segmentation module, the attention U-Net with point rend features achieved satisfactory DSC and loss in the training set, validation set and testing set, which indicated satisfactory segmentation performance. Based on segmentation, a stacked hourglass network was applied to recognize featured anatomic landmarks. The identified landmarks served as a reference for pelvis correction and recognition of preoperative deformities. The comprehensive workflow including automatic segmentation, pelvis correction, deformity assessment and real-time simulation of postoperative outcomes was integrated into the AIHIP software.

Segmentation is the foundation for building an accurate preoperative planning system. Previous studies have reported deep learning as a valuable tool for automatic segmentation in abdominal CT (12), head CT (26), and CT angiography (27). Although the role of deep learning in joint segmentation remains largely unexamined, the results of this study were comparable to the abovementioned studies. A stacked hourglass network was first used to identify featured bony landmarks in cephalograms in 2020 (28). Cephalograms are two-dimensional X-ray images, while CT offers three-dimensional information, which complicates the coordinate prediction of featured points. This study further investigated its application in hip CT. The use of artificial intelligence has also greatly reduced the time and manpower required to conduct detailed preoperative plans for patients.

In this series, AIHIP planning was significantly more accurate in predicting implant sizes than X-ray-based planning. X-ray-based planning underestimated cup size by an average of 2.13. The accuracy of X-ray-based planning varies in different studies. The reported accuracy of X-ray-based planning ranged from 40.68 to 90% (24, 29, 30). The wide variety of reported accuracies for X-ray-based planning is subjected to many factors, including the quality of the radiograph, magnification error and surgeon experience (31). In this series, the accuracy to within ± 1 size of X-ray-based planning was 55% for cup size and 65.00% for stem size, while the accuracy to within ± 1 size of AIHIP planning was above 95% for cup size and stem size. Acetate templating tended to underestimate stem size and cup size. Planners might be more conservative in X-ray-based planning because less information is provided in 2-dimensional X-rays than in 3-dimensional CT. During AIHIP planning, the simulation of prothesis position and its relation to surrounding bone can be visualized in coronal, sagittal and axial plane, allowing surgeons to adjust the plan from more angles.

Both X-ray-based planning and AIHIP planning were carried out by two orthopedic residents. This suggested the potential benefit of AIHIP in assisting beginners because it provides more information than traditional methods. In AIHIP planning, real-time simulation of the position, coverage and to what degree the deformity could be corrected were visualized, allowing for improved understanding and simulation of the case prior to surgery. AIHIP planning also facilitated accurate measurement of offset and LLD. Femoral offset was more accurately restored in AIHIP planning comparing with X-ray planning, which may be related to the fact that more high offset/varus stems were selected in AIHIP planning. It has been reported that reduced femoral offset negatively affected range of motion due to impingement and reduced abductor lever (32). Gait analysis has shown that changes in femoral offset also influence the function of external rotator, extensor and short flexor muscle during different phase of gait (33). On the other hand, one study found that significantly increased femoral offset was an important source of elevated ions in metal on polyethylene THA, suggesting possible increased contact force and accelerated wear (34). Therefore, accurate restoration of femoral offset might provide potential benefit in avoiding impingement and implant biomechanics. Although the changes of femoral offset might be compensated by changes in acetabular offset in certain aspects, a significant decrease in femoral offset might result in impingement and possible dislocation irrespective of acetabular offset. The limitations of this study are as follows. 1. Postoperative radiographic assessment was conducted on X-ray images rather than CT, which is less accurate. 2. There was no statistical significant differences in terms of clinical outcome. 3. Different planning methods were applied in the two groups, which could lead to potential bias in comparing the accuracy of both methods. However, general linear model was applied to minimize the influence of confounding factors.

## Conclusion

The use of AIHIP greatly reduced the time and manpower required to conduct detailed preoperative plans while being more accurate than traditional planning methods. It has potential in assisting surgeons, especially beginners facing the fast-growing need for total hip arthroplasty with easy accessibility.

## Data Availability Statement

Raw data of this study are deposited in the local server of our institute for safety and confidentiality purposes. The data are available for non-commercial research purposes, without undue reservation. Please contact the corresponding author (WQ) at qianww007@163.com for more information.

## Ethics Statement

The studies involving human participants were reviewed and approved by the Institutional Review Board of Peking Union Medical College Hospital. The patients/participants provided their written informed consent to participate in this study.

## Author Contributions

WQ, GQ, YZ, and XC conceived and designed the study. YZ and XL developed, trained, validated, and tested the neural networks. RM, YiW, SZ, XD, and XC conducted the radiographic measurement. XC, ShL, and SoL analyzed the data. HL, GW, and YaW checked the data and methodology. XC and XL wrote the manuscript and conducted critical analysis. All authors read and approved the final manuscript.

## Conflict of Interest

XL and YZ are employed by Longwood Valley Medical Technology Co. Ltd. The remaining authors declare that the research was conducted in the absence of any commercial or financial relationships that could be construed as a potential conflict of interest.

## Publisher’s Note

All claims expressed in this article are solely those of the authors and do not necessarily represent those of their affiliated organizations, or those of the publisher, the editors and the reviewers. Any product that may be evaluated in this article, or claim that may be made by its manufacturer, is not guaranteed or endorsed by the publisher.

## Abbreviations

THA: Total hip arthroplasty
AIHIP: Artificial intelligent preoperative planning system for THA
LLD: Leg length discrepancy
DSC: Dice similarity coefficient.
